# Health Information Seeking Behavior and Health Information Preferences Among Ethnically and Socioeconomically Diverse Patients and Communities: A Qualitative Study

**DOI:** 10.1177/21501319251332048

**Published:** 2025-05-14

**Authors:** Bryn Hummel, Dinah L. van Schalkwijk, Paula M. C. Mommersteeg, Irene G. M. van Valkengoed

**Affiliations:** 1 Amsterdam UMC, Location AMC, The Netherlands; 2 Tilburg University, The Netherlands

**Keywords:** diversity, ethnicity, cardiovascular disease, health information seeking behavior

## Abstract

**Introduction:**

Early recognition of ischemic heart disease (IHD) is important, yet, delays still occur due to low symptom recognition. Accessible information may improve symptom recognition, however, it is unclear how information should be provided to reach different populations. Hence, we studied health information seeking behavior (HISB) and preferences, in ethnically-diverse women and men in the Netherlands.

**Methods:**

We conducted 31 patients interviews, seven key figure interviews and one focus group with key figures (community leaders and physicians), and eight focus groups with non-patients (N = 44) about HISB and health information preferences. We thematically analyzed the data using inductive coding.

**Results:**

We found minimal variation in HISB, as most patients did not seek information about symptoms. Participants required information about cardiac symptoms, risk factors, when to seek care, prevention, and navigating the Dutch healthcare system. Several information provision strategies emerged, with preferences varying somewhat across ethnic groups and age groups. Ethnic minority participants described a preference for culturally-sensitive community-based live information provision. Other strategies included media, social media, and native Dutch participants mentioned public spaces and healthcare settings.

**Conclusion:**

HISB was limited in this ethnically-diverse population. Different strategies may be employed to promote symptom recognition, particularly co-created culturally-sensitive interventions.

## Introduction

Ischemic heart disease (IHD) is a condition characterized by reduced blood flow to the heart due to atherosclerosis or other coronary artery abnormalities.^1,2^ Early diagnosis and treatment is associated with better outcomes, but delays in diagnosis and care persist. Such delays are often attributed to people not recognizing symptoms and seeking care,^3^ and studies suggest that women and people of ethnic minority groups experience more delays in care.^4-7^ The high and unequal burden of IHD across population subgroups,^8,9^ also in the Netherlands,^10^ has a large economic impact.^11^ Therefore, it is important to promote early recognition of IHD to reduce these delays in care seeking.

Whether people recognize IHD symptoms as severe and/or cardiac, varies depending on the symptom presentation^3,12-14^ and patient characteristics.^15-17^ Accessible information and education on IHD symptoms could inform people about IHD symptoms and potentially reduce delays in care related to symptom recognition.^17,18^ However, literature suggests there is gender- and ethnic variation in health information seeking behavior (HISB).^19-22^ Therefore, the European Society of Cardiology highlights a need for tailored, culturally-sensitive information provision strategies to inform women and men of different ethnic groups about symptoms of IHD to reduce delays in seeking care.^2^

HISB can be defined as a process in which people seek health information, which can affect their health-related decision making and health outcomes.^23^ HISB increasingly takes place online, referred to as e-HISB.^24^ Discrepancies in HISB may in part be explained by differences in literacy, health literacy, and digital literacy,^20,25^ as well as variation in access to health information and whether available health information aligns with people’s preferences.^26,27^ When information is not accessible or does not align with an individual’s needs and preferences, they may not benefit from this information.

Currently, it is unclear whether individuals diagnosed with IHD from an ethnically-diverse populations seek information about symptoms prior to seeking care, and how information should be provided to effectively reach and inform people about IHD symptoms to promote adequate care seeking. Hence, we studied (1) whether patients sought information about IHD, and (2) preferences and needs regarding information provision about IHD, in ethnically-diverse women and men.

## Methods

### Study Design

In this qualitative study, we explored HISB, and preferences and needs concerning information about IHD among a diverse group of IHD patients. This paper builds on earlier work investigating the occurrence of – and reasons for – delays in care for IHD within the Dutch healthcare system (Supplemental Textbox 1), by studying potential solutions for the main identified issue to reduce these delays. First, in semi-structured interviews,^28^ we explored patients’ HISB and health information preferences. In key figure interviews, and focus groups with non-patients, we discussed how health information should be provided to effectively reach people of different ethnic groups, and how barriers to care could be addressed in this information.

Interviews and focus groups were led by BH and DvS, and an intern. BH has a background in interdisciplinary social sciences, and DvS has a background in medical psychology, and both had extensive training in qualitative research. We recognize the majority of our research team is native Dutch, which may have affected data collection and analysis; we will reflect on this in the discussion. We used Standards for Reporting Qualitative Research.^29^ This research was guided by a constructivist/interpretivist paradigm and employed both ethnographic and phenomenographic approaches.^30^

### Population and Sampling

Patient inclusion took place between June 2022 and December 2023. We used maximum variation sampling to enroll an ethnically-diverse group of participants, and continued data collection until data saturation was achieved, that is, additional interviews yielded no additional information.^28^ Inclusion criteria for patients were as follows: individuals residing in the Netherlands (1) between the ages of 30 to 70, (2) with a first IHD diagnosis in the past 5 years. We used a combination of purposive and convenience sampling, whereby we primarily focused on native Dutch individuals, and individuals of the largest ethnic minority groups in the Netherlands: South-Asian Surinamese, African Surinamese, Turkish, and Moroccan. In the Dutch context, ethnic origin is defined using an individual’s and their parents’ country of birth. Among ethnic minority group individuals, including migrants (first-generation, those born abroad with at least one parent born abroad) and non-migrants (second-generation, those born in the Netherlands with both parents born abroad).^31^ Participants were considered native Dutch if they, and at least on parent, were born in the Netherlands. We additionally included patients of other ethnic groups, and also aimed to include people of different socioeconomic strata (using educational level as a proxy), as socioeconomic position may also affect HISB and information needs and preferences.

Next, we conducted interviews with key figures, including community-and religious leaders, and healthcare professionals (HCPs) from ethnic minority groups or working in an ethnically-diverse setting. These interviews were used to gain insights into potential populations not reached, and gain broader perspectives on information needs and preferences among various ethnic groups.

Finally, we conducted focus groups involving non-patients from six different ethnic groups: native Dutch, South-Asian Surinamese, African Surinamese, Ghanaian, Turkish, and Moroccan. Except for native Dutch, all focus groups were natural groups (i.e., participants already knew each other). For the Turkish and Moroccan individuals, separate focus groups were held for women and men, as we found no mixed-sex natural groups for these communities.

### Recruitment

Several methods were employed to reach and recruit a diverse group of participants, including online recruitment, recruitment via HCPs, a cardiac rehabilitation center, and religious and social organizations.

### Procedure

Initial contacts with potential participants were mostly indirect, through community leaders, HCPs, and other organizations. There was no personal involvement with participants prior to data collection. Potential participants were given information forms, after which they were given some time to reflect on participation. Before the interview, participants were given the opportunity to ask any questions. Interview and focus group locations included patients’ homes, hospitals, general practice offices, religious institutions, community centers, and online.

### Data Collection

Data collection took place between August 2022 and December 2023. Interns first received interview training, prior to interviewing participants independently. Interviews were conducted in Dutch or English, and for participants not proficient in Dutch or English, we asked whether a relative or acquaintance could serve as a translator.

Participants were encouraged to share their experiences and insights freely and honestly. We used probes such as follow-up questions and brief silences to gain deeper insights. Interviews lasted between 45 min and 1 h 15 min, and focus groups between 1.5 to 2 h. Participants received a €25, – gift card as compensation for their time and effort.

### Materials

The interview guides were created by BH and IvV with input from DvS, PM, and various others, including representatives from different ethnic groups. The interview guide for patient interviews consisted of five main topics: (1) personal information, (2) symptom presentation, (3) HISB, (4) healthcare seeking behavior (discussed in a separate paper due to limited space and a different research aim^17^), and (5) interacting with HCPs (Appendix A), including questions such as: “Why did you (not) seek information about your symptoms?” As qualitative data collection is an iterative process,^28^ changes to the interview guide during data collection were discussed and logged.

In key figures interviews and non-patient focus groups, we explored how, where, when, and by whom information should be provided to reach individuals of different ethnic groups (Appendices B and C). Moreover, we discussed the barriers to care identified in our patient interviews,^17^ and how to overcome these barriers (Appendix D). All interviews and focus groups were recorded using a digital audio recording device.

### Ethical Considerations and Privacy

Our study was approved by the Amsterdam UMC and Tilburg University ethical review boards. All participants provided written informed consent and oral approval to participate in the study. Interns who supported data collection and processing signed a nondisclosure agreement.

In line with Amsterdam UMC privacy regulations, signed informed consent forms were saved in a secured cabinet at VUMC. Audio recordings were stored on secured servers at Amsterdam UMC and Tilburg University in folders only accessible to the lead researchers. Audio files and transcripts were transferred via a secured file transfer service. During transcription, the interviews were pseudonymized, after which audio files were deleted. Participant information was saved separately from transcripts, and were matched using a randomized code for each participant.

### Data Analysis

Data analysis took place between April of 2023 and April of 2024. We used qualitative data analysis software Max-QDA^32^ and Atlas.TI.^33^ We transcribed interviews and focus groups verbatim. Next, the transcripts were analyzed using the six steps of thematic analysis: familiarizing with the data, creating initial codes, searching for themes, reviewing potential themes, defining and naming themes, and writing the report.^34^ After verbatim transcription, the interviews and focus groups were coded and analyzed separately by at least two researchers: BH, DvS, or an intern. Biweekly meetings were set-up to discuss newly emerged codes in order to reach consensus between the researchers on the use and purpose of the codes. All coded transcripts were checked by a second researcher. The thematizing phase consisted of consecutive meetings discussing the content and meaning of all codes. By comparing and contrasting codes, we searched for common elements or concepts, which could be grouped together to form a theme. This was an iterative process of constant reflection and refinement to ensure that the data were properly represented and to provide a comprehensive answer to the research question.

### Quality Assurance

To ensure the validity and reliability of our qualitative data analysis, we applied several quality control measures. We applied data triangulation by interviewing individuals from a variety of backgrounds, including patients, non-patients, and professionals. With respect to investigator triangulation, at least two researchers independently coded the transcripts, and codes were discussed in bi-weekly meetings to reach inter-coder reliability. During the analysis process, interpretations and discussions were noted in an audit trail to ensure transparency. Results were shared with a diverse range of researchers (peer debriefing) to enhance reliability.

## Results

We interviewed 31 patients: fourteen women (45%, mean age 63.6 years) and seventeen men (55%, mean age 61.4 years), of twelve different ethnic groups (78% ethnic minority background, Table 1). While we aimed to include patients between the ages of 30 to 70 years, most included patients were older than 55, given that IHD is less common in younger people. Our key figures had several different ethnic and occupational backgrounds (81% ethnic minority background, Table 2). We conducted eight focus groups, totaling 44 participants: 24 women (54%) and 20 (46%) men, of six different ethnic groups (86% ethnic minority background, Table 3).

**Table 1.** Patient Characteristics.

Participant number	Sex	Age	Ethnicity	Diagnosis	Educational level^a^	Employment^b^
1	Female	65	Dutch	Coronary artery disease	Higher educated	Incapacitated
2	Male	62	South-Asian Surinamese	Cardiac infarction	Lower educated	Working
3	Male	51	South-Asian Surinamese	Myocardial infarction	Higher educated	Incapacitated
4	Male	68	South-Asian Surinamese	Acute coronary syndrome	Intermediate educated	Working
5	Male	69	Dutch	Ischemic heart disease	Higher educated	Retired
6	Male	65	Iraqi	Angina pectoris	Higher educated	Retired
7	Male	70	Dutch	Myocardial infarction	Lower educated	Retired
8	Female	68	Dutch	Myocardial infarction	Lower educated	Unemployed
9	Female	68	Dutch-Antillean (Curacao)	Myocardial infarction	Lower educated	Unemployed
10	Female	64	Dutch	Microvascular coronary dysfunction	Intermediate educated	Retired
11	Male	63	Dutch	Coronary artery disease	Lower educated	Unemployed
12	Female	65	South-Asian Surinamese	Microvascular coronary dysfunction	Lower educated	Unemployed
13	Male	66	Dutch	Myocardial infarction	Lower educated	Unemployed
14	Male	63	Turkish	Stable Angina Pectoris	Higher educated	Retired
15	Female	59	Ghanaian	Microvascular coronary dysfunction	Lower educated	Working
16	Female	70	Moroccan	Stable Angina	Lower educated	Incapacitated
17	Female	65	South-Asian Surinamese	Coronary artery disease	Lower educated	Incapacitated
18	Male	59	Turkish	Angina pectoris	Lower educated	Incapacitated
19	Male	57	Moroccan	Myocardial infarction	Intermediate educated	Working
20	Female	63	African Surinamese	Coronary artery disease	Intermediate educated	Working
21	Female	56	African Surinamese	Coronary artery disease	Lower educated	Unemployed
22	Female	73	African Surinamese	Myocardial infarction	Lower educated	Retired
23	Female	70	South-Asian Surinamese	Myocardial infarction	Lower educated	Retired
24	Male	60	Cape Verdean	Myocardial infarction	Higher educated	Working
25	Male	63	Egyptian	Myocardial infarction	Lower educated	Incapacitated
26	Male	54	Iranian	Myocardial infarction	Lower educated	Incapacitated
27	Male	50	Moroccan	Coronary artery disease	Lower educated	Incapacitated
28	Female	41	Moroccan	Coronary artery disease	Intermediate educated	Unemployed
29	Female	63	Afghan	Coronary artery disease	Lower educated	Unemployed
30	Male	62	Turkish	Myocardial infarction	Lower educated	Incapacitated
31	Male	62	Moroccan	Myocardial infarction	Intermediate educated	Incapacitated

a Educational level was defined as the highest attained educational level, either in the Netherlands or the country of origin. This was categorized into lower (including no education, elementary school, lower vocational, or secondary education), intermediate (including intermediate vocational schooling or intermediate or higher secondary schooling), or higher education (higher vocational or university education).

b Employment status was defined as people’s working status at time of the interview, and was grouped into working (those in paid employment, regardless of the number of hours worked per week), unemployed (those not in paid employment, regardless of whether they are were looking for work), incapacitated (those unable to participate in paid employment due to their health concerns), and retired (those who retired, regardless of whether they had reached the pension age of 65 or not).

**Table 2.** Characteristics of Key Figures.

Key figure number	Sex	Ethnicity	Function or employment
1	Female	South-Asian Surinamese	Health ambassador, head of a multi-ethnic foundation
2	Male	South-Asian Surinamese	Head of a South-Asian Surinamese foundation
3	Male	Turkish	Retired HCP and cardiovascular disease patient
4	Female	Ghanaian	Health ambassador, media personality
5	Female	African Surinamese	Physician
6	Male	Moroccan	Volunteer at a Moroccan Mosque and community center
7	Male	Turkish	Spiritual caretaker in a Dutch hospital
8	Male	Nigerian	Pastor of an African church
9	Male	South-Asian Surinamese	Imam of a large multi-ethnic mosque
10	Female	Dutch	Cardiologist
11	Male	Dutch	General practitioner

**Table 3.** Characteristics of Focus Group Participants.

Ethnicity	Participants	How were participants reached
Ghanaian	2 women, 6 men	Ghanaian community organization
South-Asian Surinamese	3 women, 3 men	South-Asian Surinamese community organization
Moroccan	4 women, 4 men	Moroccan student assistant, Moroccan community organization
African Surinamese	5 women, 1 man	African Surinamese community organization
Dutch	5 women, 1 man	Facebook group of a lower-middle class neighborhood
Turkish	5 women, 5 men	Turkish women’s community organization, Turkish mosque

First, we will discuss our findings concerning HISB, and subsequently, we will discuss preferences and needs in how information about IHD should be provided. Subthemes will be indicated in bold, and *sub-subthemes* in italics.

### RQ1. HISB

The first research question addressed HISB among participants of different ethnic groups. Patients were asked if, how, and where they searched for information about their symptoms before a diagnosis. Three themes emerged answering this research question.

#### HCPs as a Trusted Information Sources

Most participants, across all ethnic groups, preferred consulting their HCP, such as a general practitioner (GP) or a cardiologist when diagnosed, as their primary source of information. HCPs were seen as reliable sources of information who could give *personalized advice*. Participants preferred to rely on their expertise instead of searching for information themselves. Participants mentioned that HCPs could distribute information flyers, which would then be considered a trusted source of information.

*Int*: And you also mentioned [. . .] “that I do not seek information myself because I don’t trust it.” That is what I hear you say. Which source of information do you trust?

*Patient*: As I said, my doctors and specialists. I trust them completely.

- Native Dutch man, 66 years old.

#### Trusted Others as Information Sources

Some participants mentioned that they valued the insights and advice provided by acquaintances with medical knowledge, such as family members or friends who either worked in the medical field or had personal experience with a certain disease. Some, in particular older patients and those not proficient in Dutch, mentioned first discussing symptoms with their children and asking their adult children for advice. These patients sometimes asked their children to look up information online for them, as children were often perceived as a reliable source for retrieving and interpreting online information. Patients related this to their belief that their children generally possessed *greater Dutch language proficiency, advanced digital skills* and a *better understanding of the Dutch healthcare system* than they themselves did.

My daughters looked it up, also on the internet.

- African-Surinamese woman, 73 years old.

A small number of participants said that some individuals may consider community leaders as trusted sources of health information. They stated that some individuals with symptoms may first consult their religious leader instead of a HCP. While most ethnic minority group individuals said that community leaders may provide advice or emotional/religious support, a smaller number of people may consult community leaders for religious or spiritual healing.

#### Online Information

Contrary to our expectations, the vast majority of patients, regardless of sex or ethnicity, said they did not seek health information between symptom onset and being diagnosed. They mentioned several reasons for not doing so, including language barriers and low health literacy, that is, participants’ inability to correctly interpret health information. Patients also said the large amount of online health information caused *information* overload, and they *mistrusted or feared online sources*, as they could not always distinguish trusted health information from misinformation.

The more you read on the internet or google. . . Yes, you read everything, and I don’t know if it is true. And then I think, if something is really wrong, I am better off going to my GP. Doctor [name cardiologist] also says: “if something is wrong, just come by or call.”

- Native Dutch woman, 64 years old.

In addition, many patients stated they never considered looking up their symptoms online, for instance because they had already attributed them to other conditions (e.g., flu, gastrointestinal or other causes). Some patients sought information online after being diagnosed, including information about treatments, for instance what a percutaneous coronary intervention entails. A few patients, specifically those with greater health literacy due to having worked in healthcare, looked up information on certified platforms. Others generally used channels such as social media. Patients from ethnic minority groups mostly sought information in their native language.

### RQ2. Preferences and Needs Regarding Health Information

The second research question examines preferences and needs for information content and delivery. For example, participants were asked what information they would need to adequately seek care, and how they would prefer to receive this information.

#### Knowledge of Heart Disease and Care Seeking

Most participants mentioned that they required information about cardiac symptoms and when to seek care, in order for them to adequately seek care. Participants, including patients, often expressed a lack of knowledge and understanding about heart disease, cardiac symptoms, and potential consequences.

But what are the symptoms of the heart? No one knows that.

- Turkish men’s focus group.

Some female participants had previously heard of sex differences in cardiac symptoms, and mentioned wanting information about *sex-specific symptom presentations*.

Participants also emphasized the importance of receiving information about risk factors and prevention strategies for heart disease *tailored to ethnic groups*. This would include, for instance, sharing information about prevalence rates of cardiovascular risk factors among different ethnic groups. Similarly, participants highlighted the need for information on healthy lifestyles, including modifications to make traditional diets healthier and incorporating exercise.

So it is very important that there is more information about [. . .] it is highly prevalent in this group, you are one of them, it also happens at this age, and these are the symptoms [. . .].

- South-Asian Surinamese man, 51 years old.

Beyond information about symptoms, care seeking, and risk factors, particularly people of ethnic minority groups mentioned requiring information about the Dutch healthcare system. For instance, some participants mentioned they were not aware that general practice care is free in the Netherlands, and they agreed that information about *insurance* and *costs of care contacts* could promote peoples’ understanding of the healthcare system and reduce financial barriers to care. Moreover, many participants, including some Surinamese participants (whose second native language is Dutch), emphasized the importance of being taught how to prepare for a medical appointment, as language barriers and cultural differences in communication were frequently identified as barriers to care. Specifically, participants mentioned that the information should tackle uncertainties in *describing symptoms* and *communicating their concerns*.

Speaker 5: Oftentimes, that is the problem. Because [the older people] say they can’t express themselves, they can’t describe the symptoms well, [. . .] which means the GP often doesn’t understand them and doesn’t address it, which makes them feel misunderstood. Then they think “Well, I’m not going [to the HCP], they don’t help me anyway,” so they dismiss everything, and that is why many elderly people don’t go [to their HCP]. They haven’t learned that, what I know about [the Netherlands], you do the preliminary work before you go [to a HCP]. Speaker 2: You prepare yourself. Speaker 5: How do you prepare yourself? Just that question, when [GPs] ask, “What can I do for you?” [the older Surinamese] often don’t know how to answer that because they haven’t yet thought about what they actually have.

- African Surinamese focus group.

#### Health Information Sharing Strategies

Participants of all ethnic minority groups, but not native Dutch, expressed a strong preference for receiving information via community-based live information meetings. Participants agreed that information should be provided in an easily accessible manner, at a familiar location, to effectively reach individuals. Such familiar locations included churches, mosques, and community centers, or schools.

For instance, there is a Turkish mosque around the corner, and on Thursday morning [. . .] many Turkish women gather and have breakfast together and listen to the information they give.

- Turkish women’s focus group.

With respect to timing, participants agreed that information should be provided at a time when people are already present. Several participants indicated that they would not leave their home for just an educational information meeting, hence, information should be provided before or after standard community gatherings, such as a prayer, Quran reading, or weekly women’s groups.

All participants also mentioned flyers as a way to share information, however, they emphasized that flyers were *complimentary* to live information, not stand-alone.

I agree with [key figure 1 and 2], and I think that a flyer and such, is maybe for after a meeting, but not at the start.

- Key figures meeting.

Handing out flyers after a meeting could serve as a *reminder of the message* and could *facilitate sharing information*. All participants agreed that flyers should be easy to understand for people with lower literacy levels by making them *visual* and having flyers available in *B1-level Dutch* and *several different languages*.

Participants of all ethnic groups discussed social media as a way to share health information. While patients did not look up information online, participants mentioned that people may be reached via *different online networks*. For instance, people of most ethnic minority groups mentioned having extensive WhatsApp networks, in which information can be shared. Some participants mentioned that *video’s on social media* and collaborating with *influencers of ethnic minority groups* can be used instrumentally to inform especially the younger generations, and the messaging should be *brief and catchy*. Participants agreed that by informing younger generations of ethnic minority groups, you may indirectly reach older generations, as children often share information with their parents.

So if we also want to reach older people, then the mosque and via their children is the best way to give them information

- Moroccan man, 57 years old.

Native Dutch, Ghanaian, African Surinamese, and South-Asian Surinamese participants discussed media, such as television and radio, as a method to reach people who may not (be able to) attend live meetings, for instance due to social isolation or limited mobility. Whereas native Dutch participants mentioned *national and regional media* as effective ways of reaching native Dutch people of different socioeconomic groups, the Surinamese and Ghanaian groups mentioned having *their own radio channels*, via which information could be shared.

So if you want to reach the older generation, except for those coming to live meetings, it is always via radio shows.

- South-Asian Surinamese focus group

Lastly, primarily the native Dutch focus group mentioned public spaces and healthcare settings as a last way of sharing information, such as information screens in GP offices and pharmacies.

#### The Role of Key Figures and Role Models

Ethnic minority participants agreed on the importance of collaborating with key figures to organize live meetings and effectively reach communities. As key figures are *trusted by community members*, they play an important role in the organization of live meetings by providing an *entrance into the community*. These key figures can be anyone with a large influence in their community, for example, a pastor, Imam, or board member of a community organization.

Participants also mentioned the importance of role models. Role models could be celebrities and public figures who could help raise awareness, functioning as *health ambassadors.* Alternatively, role models could be experts by experience, that is, people of the same ethnic group diagnosed with IHD. Participants agreed that role models could *decrease stigma* on the disease, and *foster open conversations*. Participants said that for men specifically, well-known, masculine role models with whom other men could identify, could reduce barriers to care related to norms surrounding masculinity (i.e., the idea that men should be tough, and not seek care when sick).

Like that [name of famous former athlete], he also died suddenly, and people talked about it for years afterward. That was also just a young man, a healthy man [. . .].

- Native Dutch focus group.

#### Tailored, Culturally-Sensitive Information Meetings

To foster trust and promote acceptance, ethnic minority group participants emphasized the need for culturally tailored health information. Participants mentioned several ways in which information could be provided in a culturally-sensitive manner. First, during live information meetings, they agreed that information would ideally be provided by a medical professional with a similar ethnic or cultural background who *speaks attendees’ language*. This also applied to Surinamese participants, whose second native language is Dutch.

[. . .] the older Surinamese, they do not understand the [native] Dutch that well, we talk differently, we think differently, which is why a Surinamese [physician] would appeal more to us and be understood better. And they would feel safer.

- African-Surinamese focus group.

This was said to foster trust and understanding, while also reducing language barriers and cultural communication differences. Given that women and men are generally separated in Mosques, Turkish participants also mentioned a preference for a same-sex specialist.

Second, most participants stated there should be a sociable atmosphere in these meetings, for instance by providing some drinks and food. This varied somewhat amongst communities: Ghanaian and Surinamese participants deemed the sociable character of meetings very important to facilitate conversation, while this was deemed less appropriate in Mosques, according to Moroccan and Turkish participants.

[. . .] and one of the things that I used to tell my [native] Dutch friends is that food actually brings people together.

- Nigerian pastor.

Third, participants discussed storytelling and narrative information to tailor information to community preferences. Participants believed that sharing personal stories would appeal more to the audience than a formal presentation style. For instance, Ghanaian and Surinamese participants mentioned theatre as a way of providing information which *fosters engagement and understanding*, and simultaneously *reduces the taboo*. All participants agreed that information sessions should be *interactive*, including an opportunity to ask questions and have discussions. A trusted and safe environment was considered an important prerequisite for an interactive discussion, asking questions, and sharing experiences.

Participants also agreed that information should be framed positively, as this can help reduce barriers to care stemming from norms surrounding femininity (i.e., women prioritizing taking care of their families over themselves), and denial and fear (participants not seeking care due to a fear of being sick). Participants believed that *focusing on the importance of early recognition* could reduce fear and anxiety and motivate women to prioritize their own health.

It is also about how you share [the information], and I think if you put a person in front of a group and they can convey [the information] in that way, you will get through to people. You take away the fear, give them hope, and [teach them how to] recognize symptoms and when to seek care.

- Moroccan women’s focus group.

Finally, participants also mentioned the importance of paying attention to religion. Participants and key figures agreed on the importance of integrating spiritual, religious, and medical perspectives, as this could *promote acceptance of the message*.

[. . .] What is the role of the Imam, what is the role of the medical doctor, what is the role of the specialist, and how can we bring these people, this expertise, together?

- Moroccan men’s focus group.

Particularly when the message is supported by religious leaders, this can reduce barriers to care related to religious, spiritual, and alternative healing practices, as well as patient-physician sex-disconcordance. Religious leaders could emphasize that prayer and care are complementary and prayer cannot substitute healthcare, and that people should accept a HCP of the opposite sex in emergency situations. Moreover, collaborating with religious leaders and providing religious arguments in favor of seeking care can *help alleviate fear, foster hope and comfort*, and promote adequate care seeking.

#### Cardiovascular Risk Screening

Another theme that frequently emerged, in particularly among older participants, was a desire for regular health checks, similar to cardiovascular risk screening. This was deemed an effective method of *increasing awareness* and *an opportunity to share information*. A few individuals mentioned having regular check-ups, yet, they emphasized they needed to assertively ask for this. Participants stated it would be better to *actively invite people for screening* from a certain age, similar to cancer screening.

You could also send everyone a letter, like: “you turned 40 this year, it is good to have a check-up once every 5 years,” because then I would think: “oh, I will do that”. They do that for colon cancer.

- Native Dutch focus group.

Finally, participants unanimously stated that offering free health checks, such as blood pressure, blood glucose and cholesterol measurements would be a large *motivation to attend information meetings*.

## Discussion

We found that participants’ HISB was limited, and this applied to women and men across all ethnic groups. Most patients sought information with HCPs or trusted others, and almost no patients sought information about their symptoms online. With respect to health information preferences, participants indicated they needed information about cardiac symptoms, risk factors, when to seek care, prevention, and the Dutch healthcare system. Several strategies for sharing this information emerged, with an emphasis on culturally-sensitive, community-based, live, interactive information provision, in collaboration with community leaders.

One limitation to this study is related to the use of natural groups for focus groups. Whereas natural groups may promote trust amongst members and therefore foster more honest answers,^35^ these groups may also be more homogenous in their opinions, and there may be a pressure to conform to group norms.^36^ We aimed to mitigate this by encouraging discussion amongst participants, expressing that everyone is able to speak freely and voice their opinion, and by applying triangulation by interviewing a broad range of participants and professionals. Moreover, volunteer bias may have affected our findings, as we may have predominantly recruited active community members in our focus groups and interviews, and therefore, results may not transfer to less involved community members. This may also partially explain the preference for live meetings for participants of most ethnic groups, with the exception of native Dutch, as this was the only constructed focus group (i.e., participants did not know each other beforehand). In an attempt to gain insights on information preferences of less involved community members, we specifically asked participants and key figures about strategies to also reach more isolated community members.

Another limitation pertains to our methodology, as we were unable to apply certain techniques to enhance data credibility. For instance, we were unable to conduct member checking with participants due to difficulty in contacting participants over time. Moreover, some groups may be underrepresented in our study, including younger patients, meaning our findings may not be fully generalizable to all population subgroups in our study, as well as population subgroups not included in this study. This underrepresentation may partially be explained by the fact that the majority of our research team, particularly the interviewers, were native Dutch women. Sex- and ethnically-matched interviewers could reduce communication barriers and foster openness and trust when interviewing people from ethnic minority groups, which could have affected what participants may have been willing to discuss. While we invited translators to mitigate language barriers when necessary, communication barriers may have persisted and could have affected our results.

We found HISB was limited amongst people of all population subgroups, as most people did not search for information about their symptoms. In particular e-HISB was limited, as participants explicitly expressed not looking up information online, and instead preferred discussing symptoms with HCPs or trusted others. This finding contradicts earlier work, which found HISB to be common.^37-39^ The limited HISB in this study may be explained by our definition of HISB, which predominantly focused on seeking information about symptoms, versus broader definitions of HISB used in other work. Other explanations may be found in our slightly older (as most included patients were over 55 years old) and mostly ethnic minority sample,^20,38^ as HISB is more common in younger and ethnic majority populations. However, as IHD manifests itself at 67 years old for men and 71 for women on average,^40^ we may have overestimated HISB for the average patient. Our findings concerning barriers to e-HISB further align with research among African migrants in the United Kingdom, which found a high willingness to look up health information online, yet, similar uncertainties around misinformation and information overload.^41^

Despite HISB and e-HISB both being limited, participants agreed that health information can be shared directly via HCPs, social networks, and online networks. Ideally, people seek health information at HCPs, as these can immediately undertake action when necessary. However, people may refrain from contacting a HCP to discuss symptoms, if, for example, they do not recognize the potential severity of symptoms,^42,43^ particularly in non-acute situations.^17^ This emphasizes the importance of utilizing social networks for sharing health information, as people may instead seek advice, support, or information in trusted others. Sharing information via these networks may promote understanding, a sense of belonging, and reduce language barriers.^22,44^ Finally, online networks may be used to inform those active online and younger people, who may then share information with those not active online. However, while social and online networks can be used to share health information, there is also a risk of misinformation being spread through them.^45^

Participants mainly needed information about cardiac symptoms, risk factors, and when to seek care, but they also required information about prevention and the Dutch healthcare system.^46^ The need for information about symptoms and risk factors is in line with other work amongst cardiovascular disease patients,^46^ and is further exemplified by studies showing that symptom recognition is highly variable across population subgroups,^47,48^ and a frequent cause of delays in seeking care.^17,49^ In addition, our results show that this information should be tailored to the target audience, as illustrated by the need for information about sex-specific symptom presentations (e.g., symptoms like palpitations and nausea which are more common in women^50^), ethnic differences in cardiovascular risk profiles (e.g., the higher risk of IHD in South-Asian communities^51^), and the Dutch healthcare system.

Our findings indicate a need for culturally-sensitive information provision, that is, information provision which aligns with different facets of one’s culture, including language, communication, beliefs, values, rituals, and customs.^52^ This may be reflected in how, where, when, and by whom information is provided and communicated.^46^ Earlier work on underserved populations showed that information may be provided in care- and community settings, and that preferences for receiving health information vary by generation and ethnicity.^46^ Our findings partially align with these results, and we add to this work by providing more detailed information about how culturally-sensitive community-based live information provision may be implemented. Key aspects for culturally-sensitive community-based information provision include close collaboration with community leaders, a medical professional from a similar ethnic/cultural background, and providing information in community spaces. These findings align with other studies on culturally-sensitive interventions, which also found that collaboration, co-creation, and support from local community organizations are important facilitators for the effective implementation of interventions in ethnically-diverse populations.^53-55^

Whereas community-based live information provision was the foremost strategy that emerged among participants of all ethnic minority groups, this strategy was not considered effective by native Dutch participants. We hypothesize that native Dutch may have fewer regular community gatherings, and therefore, may prefer other methods of information provision. Media and social media emerged as other strategies to share information, likely due to the extensive reach of these platforms. Social media was said to be useful for reaching younger individuals of all groups, while only native Dutch, Surinamese, and Ghanaian participants mentioned traditional media as a way to spread information. This suggests that different population subgroups rely on different channels for information provision, which may result from cultural practices and the availability of – and access to – these channels.

Preferences concerning how information should be communicated can be summarized as positively-framed, visual, narrative information, in people’s native language, in which religious and cultural values and beliefs are integrated. Earlier work on culturally-sensitive interventions show that taking into account cultural- and language factors are crucial aspects for the acceptance of information in ethnically-diverse populations.^53-55^ Moreover, our findings on visual and narrative information align with studies on information preferences in ethnic minority groups.^56-58^ However, studies show mixed results regarding the effectiveness of positive (gain-framed) or negative (loss-framed) framing of health messaging,^59,60^ which is partially attributed to differences in types of health messages and audiences. Therefore, when weighing these different options, it is worth noting that the effectiveness of different public health messaging strategies needs to be determined, and beyond the effectiveness, the reach, costs, and sustainability of different information provision methods should be considered.

In addition to providing information, native Dutch participants in particular emphasized the role and responsibility of the healthcare system and GPs in proactively sharing information and identifying at-risk individuals. This is illustrated by participants voicing a need for regular cardiovascular health checks by their GP, suggesting a demand for systematic cardiovascular risk screening. However, the time- and financial constraints in Dutch general practice, and unknown (cost)effectiveness of systematic screening practices, pose significant barriers to implementing such systematic screening.^61^

## Conclusion

Overall, HISB was limited among this ethnically-diverse population. The varying health information preferences across ethnic groups suggests a need for a gender- and culturally-sensitive intervention, co-created with communities and community leaders, to improve symptom recognition^2^ and reduce barriers to care^17^ among women and men of different ethnic groups.

## Supplemental Material

sj-docx-1-jpc-10.1177_21501319251332048 – Supplemental material for Health Information Seeking Behavior and Health Information Preferences Among Ethnically and Socioeconomically Diverse Patients and Communities: A Qualitative Study Supplemental material, sj-docx-1-jpc-10.1177_21501319251332048 for Health Information Seeking Behavior and Health Information Preferences Among Ethnically and Socioeconomically Diverse Patients and Communities: A Qualitative Study by Bryn Hummel, Dinah L. van Schalkwijk, Paula M. C. Mommersteeg and Irene G. M. van Valkengoed in Journal of Primary Care & Community Health

## Appendix A. Topic List Used During Interviews with Patients

### Theme 1. Personal Information (Max 5 min)

What is your sex – age – ethnic background – highest attained educational level – employment situation – family situation – religion?

- When did you discover you had IHD?
- How was your health before the IHD diagnosis: familiar with risk factors (HT, HCL, DM)?
  - Are/were you monitored for these risk factors? Why (not)?

### Theme 2. IHD Symptoms (Max 10 min)

- What can you tell me about the first IHD symptoms you experienced?
  - Which symptoms? In which order?
  - Were symptoms chronic/intermittent/interval?
- When was this?
- During what activity did you experience these symptoms?
- What did you think caused these symptoms? When did you first suspect IHD?
- Which alternative explanation did you have?

### Theme 3. Information Search (Max 15-20 min)

If you had these symptoms now, how would you search information?

- Where would you search information? Why there?
- Which search terms?
- When would you search information? Why then?

Have you searched for information about these symptoms, when you had them?

- Why (not)? Direct reason/trigger?
- Where? Why there?
- When? Why then?
- Which search terms?
- What did you think of the information?
- Understandable information?
- What was good/could be improved?

How, when, and where should we offer information about IHD? What content? What style?

In which other ways did you seek information about your symptoms?

Which barriers did you experience, when seeking for information?

What did you need, when you were seeking for information?

### Theme 4. Seeking Medical Attention (Max 15-20 min)

Did you contact your GP with these symptoms?

- If no: where did you seek medical attention?
  - Why did you (not) contact your PG?
- When did you first contact your GP? Why then? Why delay?
  - What was the direct cause/trigger to contact your GP?
  - Important considerations to (not) go to the GP?
    ▪ Perceived risk of IHD?

Which barriers did you experience, when you first contacted the GP?

What did you need, when you first contacted the GP?

### Theme 5. GP Contact (Max 15-20 min)

What can you tell me about the contact with the GP, when u came in with these complaints?

- How did the conversation(s) go? What did you discuss?
  - How did your GP respond?
- Which actions were taken?
  - GP visits, referrals, investigations, medication?
- To what degree are you satisfied with your GP?

Which barriers did you experience, during your contact with the GP?

What did you need, during your contact with the GP

If there is time left: how do you think your GP’s sex/age/ethnic background affected how you sought medical attention?

## Appendix B. Topic List Used During Interviews with Key Figures

In your experience (as someone from [X] ethnic group, as a general practitioner, as a cardiologist), what are possible causes of *patient delays*, in a young and multiethnic population?

- Differences between women and men
- Age?
- Culture
- Communication

In your experience, what are possible causes of symptom delays?

What do you think would be a solution for these patient- and system delays?

## Appendix C. Topic List Used During Interviews with Religious Key Figures

### In the Period Between First Symptoms and the First Medical Contact

“How do delays in the care trajectory arise in women and men of different ethnic group with (ischemic) heart disease?”

*Role of Religion*

- Religious beliefs about disease and health
  - (How) could this effect seeking care
  - (How) could this effect using care
  - Treatment preferences
  - Involvement healthcare systems
- What is written in the Quran/Bible
- Religious healing over “Western” medication
- Communication between patient and HCP with different beliefs

### Possible Solution: Information About Cardiovascular Disease

“How can we reduce delays and promote adequate healthcare seeking behavior for cardiac symptoms in different ethnic groups?”

- How should information be provided
- Where should information be provided
- Who should provide this information
- How can we incorporate religion in an appropriate solution:
- What religious beliefs are important to take into account
- We want to integrate these religious beliefs, not contradict them

## Appendix D. Topic List Used During Focus Groups

### Information Cardiovascular Disease

- How can we best provide information about IHD to people of [X] ethnic origin?
  - Differences between communities? Religious versus non-religious?
    ▪ Message, language, channels?
  - How do we motivate people to go to a meetup?
    ▪ Opportunity to measure blood pressure/blood sugar
- Where should information be provided?
  - Examples of existing networks
- Who should provide information?
  - Who are trusted figures?
  - Social networks: the power of communities
- What information should be provided?
  - Information specific to [X] ethnic group?
  - How should information be framed/phrased?

### These Factors May Contribute to Delays in Seeking Care: How Can We Prevent That?

- Cultural factors
  - Taboo on disease
  - Language and communication barriers
  - Ethnic care (medicinal herbs): integrating western and traditional care
  - Sharing concerns with family first?
- Religion
  - Praying for a cure instead of seeking care
  - Disease as a punishment
  - Physical examinations and a doctor of the opposite sex?
- Gender-related factors
  - Women prioritize family over themselves
  - Masculinity and not seeking care/Men prioritize work
- Socioeconomic factors
  - Financial concerns trump health
  - Fear of high medical costs (insurance)
- Healthcare system related factors
  - Not feeling heard
  - Mistrust Dutch medical system
- Other
  - Fear of being sick

## Statements and Declarations

**Ethical Consideration:** The study was approved by the Medical Ethical Review Committee at the Amsterdam UMC (reference W21_544 #21.600) and Tilburg University Ethics Review Board (reference TSB_RP647).

**Consent to Participate:** Interns who supported data collection and processing signed a nondisclosure agreement. All participants provided written informed consent, and oral approval to participate in the study and record the interview prior to enrollment in the study.

**Data Availability:** The raw/processed data required to reproduce the above findings cannot be shared at this time due to legal/ethical reasons.
