# Supplementary material for: Health Information Seeking Behavior and Health Information Preferences Among Ethnically and Socioeconomically Diverse Patients and Communities: A Qualitative Study
Source: J Prim Care Community Health. 2025 May 14;16:21501319251332048. doi: 10.1177/21501319251332048 (PMC12078987; doi:10.1177/21501319251332048)
Supplement: sj-docx-1-jpc-10.1177_21501319251332048 – Supplemental material for Health Information Seeking Behavior and Health Information Preferences Among Ethnically and Socioeconomically Diverse Patients and Communities: A Qualitative Study [file sj-docx-1-jpc-10.1177_21501319251332048.docx]

**Supplementary Textbox 1. Dutch context and healthcare system**

The Netherlands has universal healthcare with mandatory basic health insurance for all residents over the age of 18^1-3^. All insured adults pay a monthly health insurance premium, in addition to a yearly deductible, i.e. the costs that an individual pays themselves before insurance covers further costs^2,3^. For individuals who cannot afford basic health insurance, there is a healthcare benefit available to help cover these costs^1^. This basic health insurance covers all essential care, including GP visits, diagnostics, specialist visits, hospital admissions, and some medications^1,2^ after the yearly deductible has been met, with GP visits as the exception. Patients do not pay for GP consultations, regardless of whether they have met the yearly deductible.

The Dutch healthcare system consists of three tiers: primary, secondary, and tertiary care^3^. Primary care comprises general practice care, and secondary and tertiary care is covered by emergency care, general hospitals, academic hospitals, and specialized hospitals or clinics. All individuals residing in the Netherlands are expected to be registered with a GP. To ensure the accessibility and affordability of care, the GP is generally a patient’s first care contact, and functions as a gatekeeper for secondary and tertiary care, as GPs may decide to refer patients to a specialist^1,2^. The exception to this rule is in emergency situations, since people may call emergency medical services instead of getting a referral through a GP.

1. Ministry of Health Welfare and Sport. *Healthcare in the Netherlands*. 2016. <https://english.zorginstituutnederland.nl/about-us/publications/publications/2016/01/31/healthcare-in-the-netherlands>

2. Kringos DS, van Riet Paap J, Boerma WGW. The Netherlands. In: Kringos DS, Boerma WGW, Hutchinson A, Saltman RB, eds. *Building primary care in a changing Europe: Case studies*. World Health Organization. Regional Office for Europe; 2015.

3. Daley C, Gubb J, Clarke E, Bidgood E. *Healthcare Systems: The Netherlands*. 2013. <http://www.digitalezorg.nl/digitale/uploads/2015/03/netherlands.pdf>
